# The Southern Dental Association

**Published:** 1872-09

**Authors:** 


*The Southern Dental Association.*—The fourth annual meeting of this Association, held in Richmond, Va., last month, was one of the most interesting we have ever attended, being characterized by that fellowship and harmony which are so necessary for the strength and support of all organizations having for their object general improvement.

The members of the Virginia State Dental Society were untiring in their efforts to entertain the Association; and they succeeded in making all their guests feel as if they were among brethren who were actuated by a sincere desire to render their stay among them pleasant and agreeable. The full proceedings of the meeting are published in the present number of the JOURNAL, and will prove interesting and instructive to those who were prevented from being present.

The next meeting of this Association will be held in Baltimore, commencing on the last Tuesday in July, 1873, when we anticipate a large and interesting meeting.

